# Mr. Mantal, in Reply to Dr. Kinglake, on Gout

**Published:** 1805-11-01

**Authors:** 


### *To the Editors of the Medical and Physical Journal.*

GENTLEMEN,

THE unsettled and indeterminate notions that prevail, concerning the nature of gout, are too obvious not to have fallen under every one's observation. This variety of opinions affords a presumptive proof, that there is not one, upon which we can confidently rely, as on a certain guide to any consistent, rational, and uniform practice. When Dr. Kinglake's Dissertation, with the annexed cases, appeared, I was sanguine enough to hope that a considerable progress was made towards a right understanding of this complex disorder. The great simplicity both of his theory and mode of treatment was,

 indeed, indeed, a strong recommendation of both; but I could not persuade myself that it exactly corresponded with the various and Proteus-like appearances, exhibited by gout in different constitutions. Cold water would effectually remove, I could easily believe, excess of temperature; and if not contra-indicated by systematic torpor, so common an affection in gout, deserved the character of a *specifick remedy* for the inflammatory stage of that disorder. What appeared to me the principal defect in Dr. Kinglake's doctrine, I endeavoured to supply by a theory, that did not pretend to originality. It is more properly a modification of the theories of Cullen and Darwin. It directs the principal attention to the constitutional affection, of which Dr. Kinglake's theory takes little or not any notice. It attempts to reconcile preceding theories to a novel practice, which at that period had proved uniformly successful; of which indeed, there was not a single instance of failure on record, though experiments had been made to a large extent by numerous practitioners in different parts of the kingdom. Its principal proposition is, that cold water will never prove injurious in gout, but from its incautious and too early exhibition; that is, under circumstances of systematic torpor, while the connection may be supposed to subsist between the torpid stomach and inflamed extremities. To confirm me in this opinion, three cases, communicated by persons with whom I had no previous connection, have appeared; and I have pointed them out, as corroborative of my theory, and to deter others from a practice, which, under similar circumstances, may be expected to produce similar bad effects: though I am sensible, that a greater number of cases, and further observations are necessary, before it can be regarded as a certain truth. I took the opportunity, as occasions presented themselves, of remarking with freedom on the peculiar sentiments of Dr. Kinglake, on the subject of gout; and, indeed, he has returned the compliment with interest, in his observations on my sentiments respecting the same subject, in the 79th number of your Journal. It is my intention, with your leave, to reconsider the question between us; to examine the arguments urged by Dr. Kinglake, as well *against* the theory which I have imbibed, as in defence and confirmation of *his own*.

It was a real gratification to me to be told, that my former remarks did not at all "displease him;" and I sincerely hope that the further observations, which his reply has induced me now to make, may meet with the same good fortune.

The

The too ardent pursuit of an argument may probably lead me, as it has led Dr. Kinglake, to adopt epithets, which, if taken separately and unconnected with the argument, would be offensive to the ear, but which their dependance on the argument explains, and in some degree justifies. This I mention as a general apology, which I am desirous to make, and am willing to accept, on all such unpleasant occasions.

I had called that part of Dr. Kinglake's theory, which confines gout to the ligaments and tendons, "a gratuitous assumption. Dr. Kinglake espoused this opinion, contrary to the generally received belief; he was the first who taught that gout was exclusively a disease of that structure; he erected on this foundation a perfectly new and most important corollary, the impossibility of visceral gout. It was not. therefore, too much to expect, that he would *demonstrate* the truth of a position, from which he deduced so important a conclusion. If he failed in this respect, what was the position but "a gratuitous assumption" to the extent of his failure in that requisite proof? Is there no possibility of bringing *demonstrative* evidence to decide this question? Dissections have made us acquainted with several diseases that are peculiar to the tendons and ligaments; have taught us that sprain is a disorder of that *whole* structure; that one species of white swelling is a disease of the ligaments only, and not of the tendons. But dissections have given us no information respecting the parts inflamed in gout. Demonstration fails; Dr. Kinglake is obliged to *infer* the fact, by asking, "if every feeling, and every external appearance in gouty inflammation do not concur to prove, that the affection originates and chiefly continues on that particular structure?

It is perhaps impossible, *from the feeling*, to discriminate with certainty, whether the pain proceeds from the joint itself, from the ligaments and tendons, or from the adjacent membranes. And if it could be discriminated, it would be of little use in determining the part affected; for the seat of *pain* is not always the seat of *disease*: as in white swelling, an acute pain extending along the tendinous aponeuroses of the muscles is said to be a concomitant symptom; but dissections prove that the ligaments only are diseased: And the pain is felt at the end of the urethra, but the stone which occasions it, stimulates the neck of the bladder. The *feeling*, therefore, is a fallacious test in favour of Dr. Kinglake's hypothesis; but not more fallacious than the "external appearance," to which he appeals peals with equal confidence. To the view, gout exhibits a shining, florid, tumefied *surface*; the part affected coloured with a *deep redness*; the inflammation apparently raging on the skin; the effusion into the adjacent cellular substance. The *vessels of the skin* and cellular membrane, therefore, in *external appearance*, are the seat of inflammatory gout.

But it should seem, that the appearance of the *local* disease was not so much in Dr. Kinglake's contemplation as a *general view* of the symptoms, as they affect the system; for in stating this argument, he professes to give *a general view* of the accession, progress, and termination of a gouty paroxysm. But, surely, "aliquando bonus dormitat Homerus." Is it forgotten, that it is the principal peculiarity of his theory, that gout is merely a *local*, and *not* a constitutional disease? But what are the enumerated symptoms of this local or otherwise constitutional disease? "Rigor, increased heat, indigestion, loss of appetite, bilious tinge of the secreted fluids, head-ach, and the usual oppressive sensations of symptomatic fever." These symptoms evincing conjointly an affection of the whole system, by what ingenuity of argument they can be made to prove that gout is not only a *local* disorder, but a disorder of the ligaments and tendons *only*, I freely confess, entirely surpasses my comprehension. These symptoms are said to be "induced by the tardy accession of inflammatory disease on the ligamentous and tendinous structure," or "before the latent inflammation assumes its formal character;" that is, to precede the gouty paroxysm. But is this a correct, full, and impartial enumeration of preceding symptoms? Our most distinguished master in the School of Medicine would hardly guess that it was meant to describe the accession of that disorder, the principal signs of whose approach are, according to the experience of that accurate observer, "an unusual coldness of the feet and legs, a frequent numbness alternating with a sense of prickling along the whole lower extremities; the body affected with some degree of torpor and languor; the functions of the stomach particularly disturbed; with the commencement of the painful paroxysm, there is commonly more or less of a cold shivering, which, as the pain increases, gradually ceases, and is succeeded by a hot stage of Pyrexia." First Lines, 506, 507, 509. This description (and its justness, proceeding from such high authority, will not be rashly called in question) much better agrees with the theory of *a torpor of the vital organs*, than with that which which Dr. Kinglake contends for. It is not denied, that considerable fever sometimes precedes a paroxysm; it is one of those characteristicks, by which Heberden distinguishes gout from rheumatism; (Comment. p. 56.) but it is obvious, on my principles, to attribute its occurrence to the reacting of the vital power, subsequently to the torpor of the vital organs.

Dr. Kinglake's last argument for the tendinous and ligamentous inflammation remains to be examined, the analogy of common sprain to gout. He seems to consider those disorders, as giving occasion to similar effects on the system; that after the accident, which produces sprain, some hours elapse before inflammation supervenes; that during its latent formation, sympathetic commotion is generated throughout the system, which in some degree subsides, when the local inflammation is fully formed. As similar phenomena are said to appear in gout, it is concluded, What? Why, that sprain and gout are the same disorder; namely, an inflammation of the ligaments and tendons: their only difference consisting in their production from a different cause; the inflammation of the ligaments and tendons in sprain arising from mechanical violence; and the inflammation of the ligaments and tendons in gout, from a "*distempered state of systematic* excitability, preponderating on the ligamentous and tendinous structure."

But, first, I deny that the sympathetic commotion generated in sprain at all resembles that constitutional affection, which occurs previously, and sometimes subsequently to an attack of gout. Its *previous* occurrence in ordinary cases is comparatively trifling, and takes place *before there is the least ground* to suspect an inflammation of the extremities; "for several days, sometimes for a week or two, before a paroxysm comes on." First Lines, 507. Its *subsequent* occurrence, unless it be speedily removed by the most prompt and the most stimulant remedies, (the effect of stimulant remedies in its removal is a strong reason for believing that it is an *affection of torpor)*, quickly puts a period to life. Instead of subsiding, as in sprain, when the inflammation is fully formed, the inflammation in gout, that had been formed, (but in general not *perfectly* formed) subsides upon the accession of the internal affection; evincing, on the principles of my theory, a deficiency of the vital power, an inability to support that *salutary* inflammation on the extremities, which in common cases excites so much action and sensation, that a portion, portion, sufficient to remove the internal torpor, is sympathetically propagated over the system.

But, secondly, even granting a similitude of constitutional ailment in the two disorders, the commotion of the system in sprain is explicable upon a very different principle. The balance of the moving powers is suddenly destroyed by the *injury that is sustained.* The inflammation in the mean time slowly proceeds; and though it is a departure from the just standard of equal action, which perfect health requires, yet the very slowness of the inflammatory accession affords an opportunity for nature to accommodate herself to the change; for the balance to be gradually restored; for the vital action to return to the parts, which it had partially deserted. And thus, agreeably to Dr. Kinglake's remark, "when the inflammation is fully formed, the sympathetic commotion in some degree subsides.

It is, perhaps, of no consequence, even if it were possible, to determine the *nature* of the spirit of animation, or the vital power. We can only judge of it from its sensible effects. It may be "a certain portion of excitability, assigned to every being at the commencement of its living state," according to Brown. Or, it may be "*a subtle material,* capable of transmission through the minutest vessels of an animal body; an *etherial fluid,* separated from the blood by the brain, and perpetually dissipated in the actions of the muscles and organs of sense," according to Darwin. Or it may be something, very different from either of those suppositions. Whatever it is, the *fact* is evident to our senses, that when any part is violently disordered, the *system* is sure to participate in the mischief; the vital power seems to be occupied at the post of danger, and other parts to be deserted. When a violent blow is received on the head, when an artery is divided with considerable loss of blood, when a nerve is wounded, when any great contusion happens, *paleness of the skin, sickness of stomach,* and a general fit of *shaking,* are not unusual symptoms. The constitutional ailment sometimes occurs immediately, at other times *after some delay;* but always evinces a deficiency of the vital power in the system, while its *quantity* seems to be increased, or its *quality* to be heightened, in the part that has been violently hurt. The agreement between the mind and the body, has often been remarked. Something similar to what has just been observed respecting the body, takes place in the mind, when it is influenced by the *ruling passion.* Whether it be ambition, ambition, avarice, anger, love, jealousy; whichever predominates in the mind, very soon engrosses and absorbs all the other affections. Thus the Poet,

——— "One master passion in the breast,
"Like Aaron's serpent, swallows up the rest."

The ruling passion is the mind's disease, which draws and converts all the faculties into its own nature.

"Each vital humour, which should feed the whole,
Soon flows to this, in body and in soul." POPE.

( To be continued. )

